# CBA/CaJ mouse ultrasonic vocalizations depend on prior social experience

**DOI:** 10.1371/journal.pone.0197774

**Published:** 2018-06-06

**Authors:** Kali Burke, Laurel A. Screven, Micheal L. Dent

**Affiliations:** Department of Psychology, University at Buffalo, SUNY, Buffalo, New York, United States of America; University of Missouri Columbia, UNITED STATES

## Abstract

Mouse ultrasonic vocalizations (USVs) have variable spectrotemporal features, which researchers use to parse them into different categories. USVs may be important for communication, but it is unclear whether the categories that researchers have developed are relevant to the mice. Instead, other properties such as the number, rate, peak frequency, or bandwidth of the vocalizations may be important cues that the mice are using to interpret the nature of the social interaction. To investigate this, a comprehensive catalog of the USVs that mice are producing across different social contexts must be created. Forty male and female adult CBA/CaJ mice were recorded in isolation for five minutes following either a one-hour period of isolation or an exposure to a same- or opposite-sex mouse. Vocalizations were separated into nine categories based on the frequency composition of each USV. Additionally, USVs were quantified based on the bandwidth, duration, peak frequency, total number, and proportion of vocalizations produced. Results indicate that mice differentially produce their vocalizations across social encounters. There were significant differences in the number of USVs that mice produce across exposure conditions, the proportional probability of producing the different categories of USVs across sex and conditions, and the features of the USVs across conditions. In sum, there are sex-specific differences in production of USVs by laboratory mice, and prior social experiences matter for vocalization production. Furthermore, this study provides critical evidence that female mice probably produce vocalizations in opposite-sex interactions, which is important because this is an often overlooked variable in mouse communication research.

## Introduction

Many animals rely on acoustic communication for transferring important social or environmental information from a sender to a receiver [1]. In many species, signals vary with both environmental and behavioral contexts, in order to convey information about species and kin identification, hierarchical status, and sexual attraction [1]. Mice have emerged as a valuable model to study acoustic communication and communication disorders, since they produce ultrasonic vocalizations (USVs) in a variety of circumstances. Recent work shows that USVs are produced in context-specific ways (e.g., [2]) and there is evidence that USVs can be used to convey individuality and kinship [3]. The research on context specificity has implied that USVs are important for the mouse courtship sequence [4–5], with particular vocalizations being emitted during specific stages of the mating sequence [2,6].

Mouse vocalizations have variable spectrotemporal features [7–10]. Researchers use the frequency characteristics of USVs to parse them into different categories, with the number of categories ranging from three to twelve across studies [7,11–15]. Although categories are found throughout the literature, it is possible that mice would not use the same criteria to divide USVs into distinct vocalization types. Researchers suggest simple spectrotemporal similarity is enough for discriminating between vocalizations [16].

There is a genetic component to ultrasonic vocalization production, suggesting that a catalog of USVs produced by commonly used strains is critical to evaluate normal vocal behavior. Strain-specific differences have been discovered in USV production [13,16–21]. Neural recordings in the auditory midbrains of mice to mouse USVs also differ across strains [22–25]. The acoustic differences in USVs across strains are behaviorally discriminated by mice, with females showing a preference for USVs from a strain that is different from their own [26].

The social and environmental context that a mouse experiences also directly affects vocal production. C57BL/6J male mice produce vocalizations which have longer durations, and they produce them more frequently, in novel environments than in familiar environments [27]. Male CBA/J mice change the dominant frequency, duration, and bandwidth of their USVs during courtship in response to relevant social information such as the presence or absence of a female and the sexual receptivity state of the female [12]. Food restricted female NMRI mice produce more USVs in the presence of a mouse that has recently eaten, regardless of the quality of the food eaten by the partner [28]. Male C57BL/6J mice will reliably vocalize when paired with other females, when presented with the urine or saliva from a female mouse, and when presented with a swab of vaginal fluid [29–32]. Although male mice will produce vocalizations to various stimuli from females, male wild house mice produce fewer USVs in response to frozen female urine than fresh urine [33]. Twelve different strains of female mice reliably vocalize to other females that they can see and smell, as well as to anesthetized females [31,34–35], and the number of USVs produced by these females is comparable to the number of vocalizations produced by males [32]. In sum, while the recording procedures and stimuli used to elicit vocalizations from mice vary across studies, it is clear that the social context leads to differences in vocal production in mice.

Sex differences in USV production have also been reported in some strains of mice. For example, female C57BL/6NCrl mice will vocalize more often to a female intruder than males will vocalize to a male or female intruder [36]. Hormones in males [37] and pheromones of females [38–39] also play a role in facilitating vocal behavior, with males vocalizing more when they are sexually mature and females producing the most vocalizations when they are sexually mature but not sexually receptive [35]. While males will vocalize to anesthetized females [40], females do not vocalize to anesthetized males [34]. These differences between males and females could imply that the sexes need different sources to motivate them to vocalize. Sex differences are not observed in all strains, however. In wild California mice (*Peromuscus californicus*), for example, males and females produce similar vocalizations in similar contexts [41], and Hammerschmidt [36] found similar acoustic features in the vocalizations of male and female C57BL/6NCrl mice. Thus, some contexts may require different vocalizations from males and females, while other contexts may not.

One of the main goals of the current study was to determine whether there are sex differences in vocalizations produced by CBA/CaJ mice in different contexts. Historically, it has been suggested that females do not contribute to the USVs recorded during courtship and mating (e.g. [40]), despite both males and females producing USVs in a variety of social situations [2,42–43]. Early studies on mouse vocalization production concluded that female mice do not vocalize to an anesthetized or devocalized male mouse [40,44–45], which led to the erroneous assumption that females will not vocalize at all during male-female interactions. These older studies, in combination with difficulty in precisely pinpointing which mouse is vocalizing during a dyadic interaction, has led researchers to attribute most vocalizations recorded from mixed-sex dyads to the males (e.g., [4]). Recently, researchers have found ways to determine which mouse in an interaction is producing USVs by using microphone arrays (e.g., [5]). This technique has improved localization of vocalizers to just a few degrees [8]; however, the single most effective way to know with absolute certainty which mouse is vocalizing is to record from mice while they are in isolation.

It is clear that there is a need for a comprehensive context-specific catalog of USVs for both sexes of mice. Another aim of the current experiment was to investigate USV production by male and female CBA/CaJ mice immediately following a period of social interaction. To avoid the problem of not knowing which mouse is vocalizing in a dyadic interaction, all mice were recorded in isolation. Recordings followed one of three social exposure conditions: non-exposure, same-sex, and opposite-sex. We hypothesized that USV production rate would not differ between males and females, as was generally found by [36] for the C57BL/6NCrl strain of mouse. Previous studies (e.g., [35]) showed that sexually receptive females vocalized less than non-sexually receptive females to conspecific female intruders, therefore all mice were recorded only when in diestrus, the non-sexually receptive state. We also predicted that the spectrotemporal features of bandwidth, duration, and peak frequency might vary across exposure conditions, providing further evidence that USVs are context and/or sex specific.

The design of this study captures USV production by mice in isolation following different types of social exposures. The differences we found in the production of vocalizations under isolation can only be attributed to the prior social exposure or isolation conditions. If the USVs were recorded from dyads of mice, it is likely that there would be even greater differences in the vocalizations. Specifically, the effects of caller sex and exposure condition on the number of vocalizations, distribution of syllable types, and spectrotemporal features might be even more pronounced if the recordings were to take place *during* the various social interactions instead of *after* they concluded. Nevertheless, this study provides evidence that researchers must consider the experiences of their mice before the recordings occur in order to fully understand a mouse’s vocal repertoire.

## Materials and methods

### Ethics statement

All procedures were approved by the University at Buffalo, SUNY’s Institutional Animal Care and Use Committee (IACUC) under protocol PSY13056N.

### Subjects

**Fig 1:**
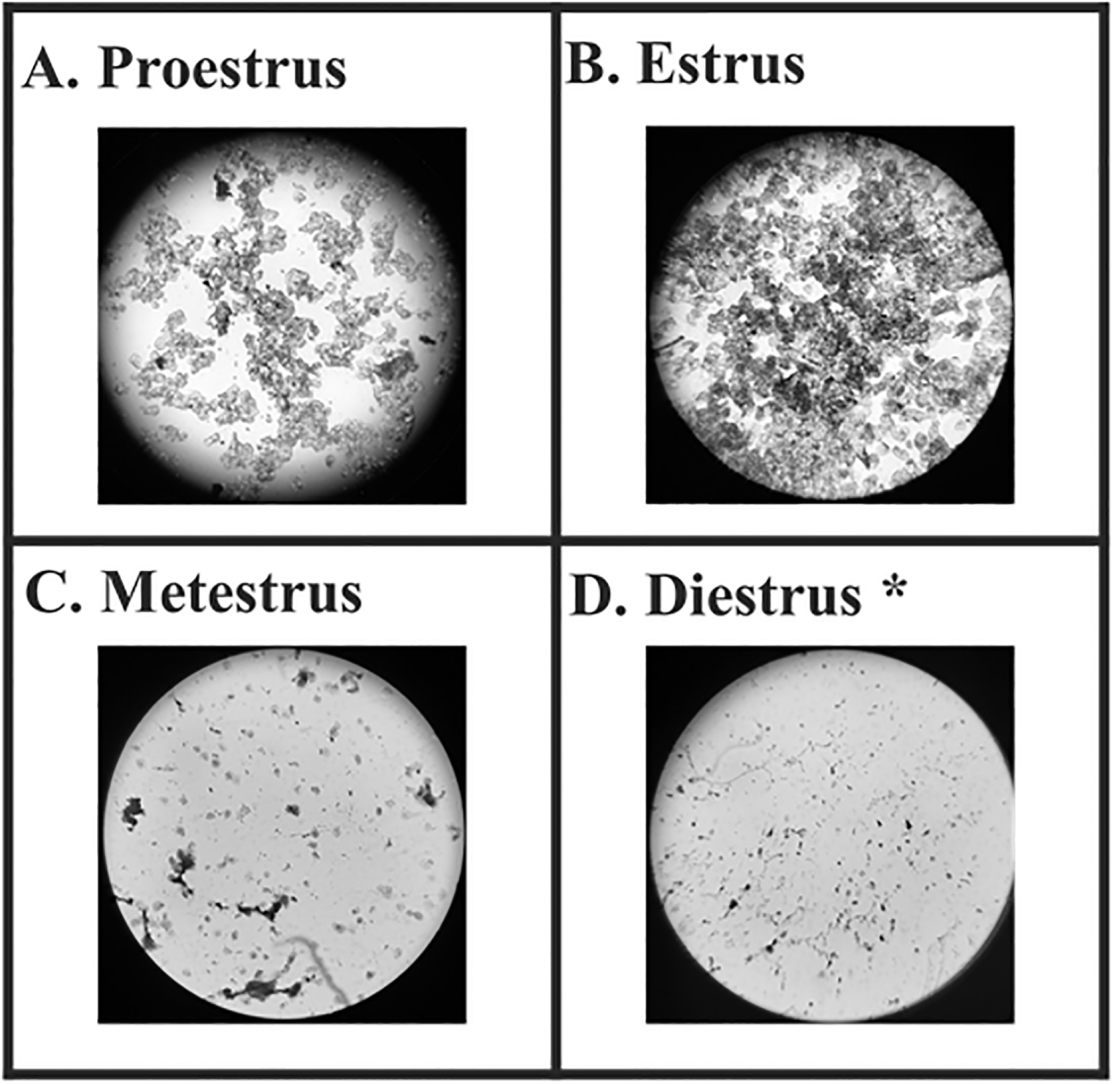
Sample slides showing vaginal smears dyed with crystal violet to represent the four different stages of the estrous cycle. The * indicates the diestrus phase of estrus, the physiological state of all females during recordings.

A total of 40 CBA/CaJ (20 male and 20 female) mice ranging in age from 10 to 17 months were subjects in this experiment. Female estrous cycles were monitored using vaginal cytology by examining vaginal wall cells for the presence or absence of leukocytes, cornified epithelial, and nucleated epithelial cells (Fig 1) (see [46] for full methodology). Females were only recorded during the diestrus phase of the estrous cycle. Estrous cycle phase tracking was completed every day throughout the course of the experiment due to irregularities in the progressions of the cycles in some females.

All experimental mice were housed individually post-weaning in a vivarium at the University at Buffalo, SUNY, and kept on reverse day/night cycle (6 A.M.–lights out, 6 P.M.–lights on). All mice were recorded during the dark portion of their cycle under red light conditions. All mice had food and water *ad libitum* throughout the duration of the study except during the 1-hr social exposures and 5-min subsequent recordings. The original breeding mice were obtained from the Jackson Laboratory, and all experimental mice were bred at the University at Buffalo, SUNY.

### Exposure apparatus

**Fig 2:**
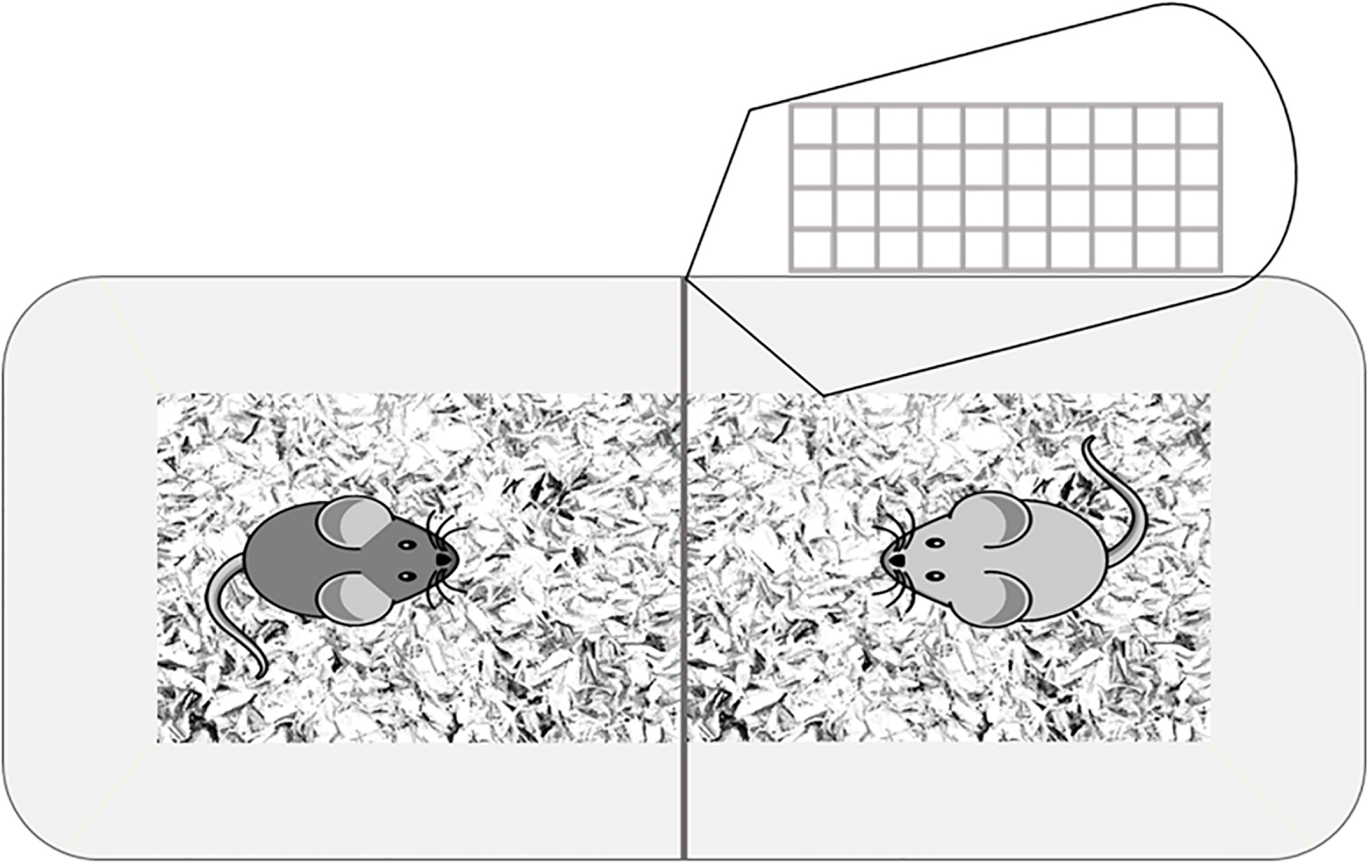
Exposure apparatus. The exposure apparatus was a standard mouse cage lined with wood shavings, divided in half with a metal mesh divider fixed to the cage. Mice were placed in this cage for one hour prior to recordings.

During the exposure phase, the recording same- or opposite-sex mice were placed together into a cage for one hour. Both of these mice were then used for recordings, and the recording of both mice took place at the same time. Mice in the non-exposure condition were placed in the exposure cage alone for one hour prior to recording. The exposure cage was a standard mouse cage (30 x 19 x 13 cm) lined with wood shavings, divided in half with a metal mesh divider (19 x 13 cm) fixed to the cage (Fig 2). The exposure apparatus was designed to allow sensory cues to be shared between the two mice while preventing the mice from mating or fighting.

### Recording apparatus

**Fig 3:**
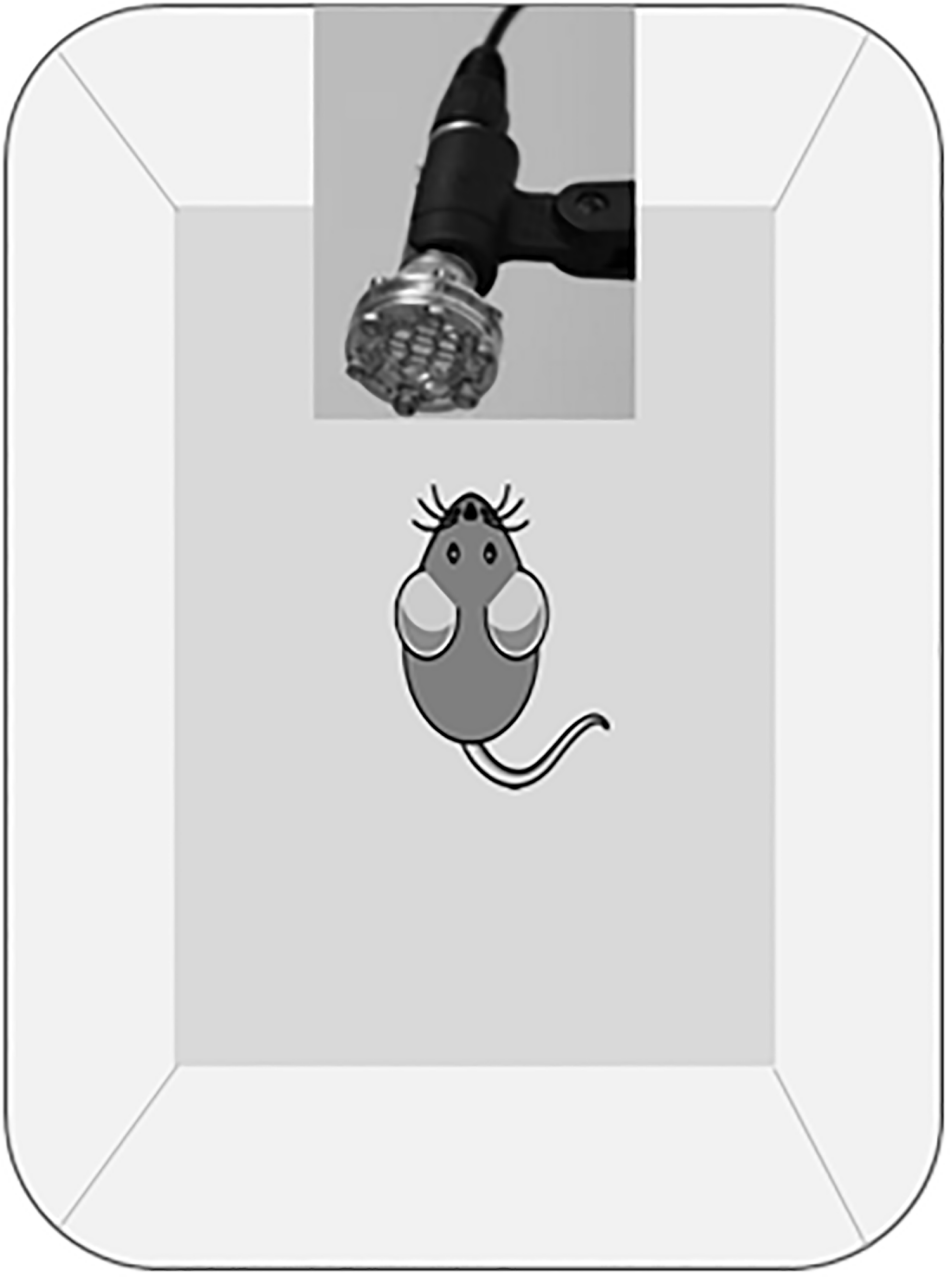
Recording chamber. The recording chamber was a standard mouse cage lined with a paper towel to reduce noise. The recording cage was placed into a homemade double walled sound attenuated booth lined with anechoic foam. An ultrasonic condenser microphone was placed above the recording cage pointing downwards. Mice were recorded in this chamber in acoustic isolation for five minutes.

The recording chambers were standard mouse cages (30 x 19 x 13 cm) with a paper towel lining the bottom to reduce noise generated by the mouse moving around (Fig 3). These cages were placed inside a double-walled sound-attenuated booth lined with anechoic foam (10.2 cm Sonex, Illbruck Inc., Minneapolis, MN). Ultrasonic condenser microphones (Avisoft Bioacoustics, model CM16/CMPA, Berlin, Germany) were placed above the recording cage pointing downwards, about 50 cm from the bottom of the cage.

The mice were recorded using an Avisoft UltraSoundGate recorder (UltraSoundGate CM16/CMPA, flat frequency response (± 6 dB) between 25 and 140 kHz) connected to a Hewlett Packard (HP) Notebook and Avisoft-RECORDER software.

### USV recordings

All mice participated in four recording sessions: one following an opposite-sex exposure, one following a same-sex exposure, and two sessions following non-exposure. Recording sessions were separated by 1 to 10 days (depending on the estrous cycle) for each mouse. The length of the estrous cycles likely varied across subjects because of the individualized housing conditions. The length of time between recordings was not included as a factor in the analysis. Each mouse completed all three conditions in a random order, with a different random order generated for each subject. Every mouse was recorded for a total of five minutes per session, immediately after being placed in the recording chamber. Similar to previous experiments in which researchers recorded from mice immediately after placing them into a novel environment (e.g., [43]), no habituation period was used in order to capture all post-exposure vocalizations. In their study, Chabout and colleagues only recorded animals for four minutes since production of vocalizations stops after a short period of time [43]. In support of this, we also found that no animals vocalized past four minutes.

### USV analysis

USV extraction and analysis were conducted using Raven Pro (v. 1.5, Cornell Lab of Ornithology). The classification of all vocalizations was completed by eye, using two raters. Extraction was accomplished through visual inspection by investigators KB and LS. Nine USV categories (chevron, chirp, complex, downsweep, flat, harmonic, inverse chevron, jump, and upsweep) previously described [2,7,12] were used in this study. A USV was categorized as a chevron when the USV increased in frequency with the highest frequency reaching >5 kHz above the beginning and end frequencies. Chirps were short USVs less than 10 ms in duration. Complex vocalizations were USVs that contained two or more directional changes in frequency and >5 kHz modulation of frequency. Downsweeps were vocalizations that started at a higher frequency than they ended (with the frequency change greater than 5 kHz). Flat vocalizations had less than 5 kHz of frequency modulation. Harmonic vocalizations were USVs containing at last one segment with at least one harmonic. Inverse chevron vocalizations decreased and then increased in frequency with the lowest frequency reaching >5 kHz below the beginning and end frequencies (shaped like a U). Jump vocalizations contain at least one break in frequency with no break in intensity and no harmonics (sometimes there were multiple jumps within one USV). Upsweeps were USVs increasing in frequency (with the frequency change greater than 5 kHz). All categories of USVs were modified from [12]. Scoring of USV recordings used a minimum interrater reliability criterion of 95%. The number of vocalizations were obtained for each subject in each exposure condition, and then the bandwidth, duration, and peak frequency were extrapolated for each USV in each category. Mean results for the spectrotemporal characteristics of each subject’s USVs in each category are presented.

### Data analysis

The vocalizations produced by mice were not normally distributed, so non-parametric tests were conducted. The Kruskal-Wallis test was used to analyze USV production, features, and proportional production of vocalizations in different categories. Significant main effects were further analyzed using Mann-Whitney U t-tests. A Wilcoxon sign rank test also used to evaluate differences in the number of calls produced between males and females. USVs were analyzed for differences across males and females in each of the three exposure conditions (Kruskal-Wallis). Next, the proportional production of each type of USV was evaluated within the sexes and across conditions for each category separately using Kruskal-Wallis tests. Finally, a feature analysis was conducted separately by vocalization category (e.g., “chirp”) for each feature (e.g., “bandwidth”) using Kruskal-Wallis tests. All significant main effects were further analyzed with Mann-Whitney U t-tests. Results of comparisons for proportional production and feature analysis were considered statistically significant with *p* values of .017 or below to reduce type I errors.

## Results

### Number of vocalizations produced

**Fig 4:**
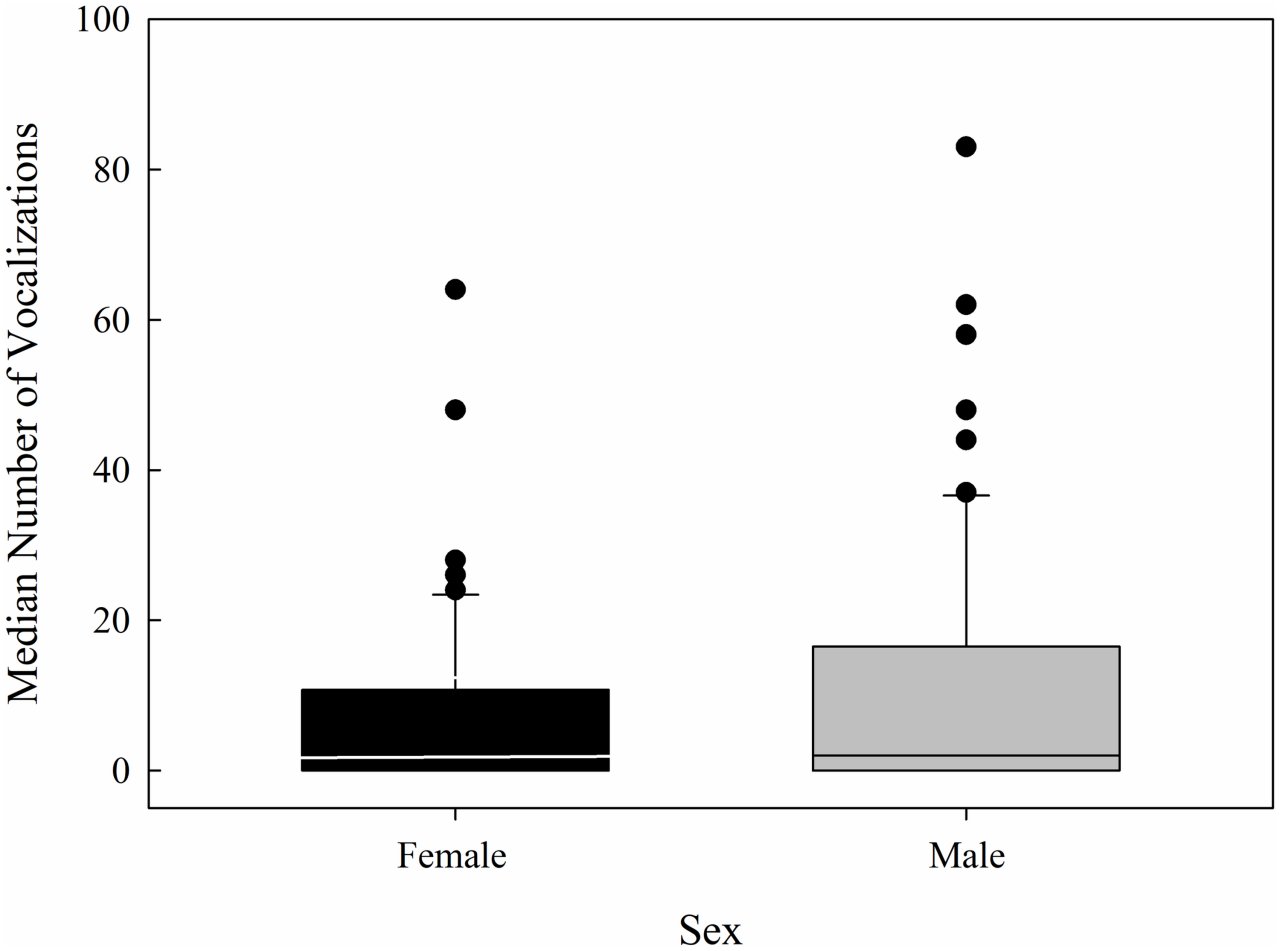
Number of vocalizations across the sexes. Box plot showing the range of vocalizations produced by males and females, the median (line in the box), and 95% confidence intervals. The black dots represent data points that lie outside the 10^th^ and 90^th^ percentiles. Males are gray, females are black.

**Fig 5:**
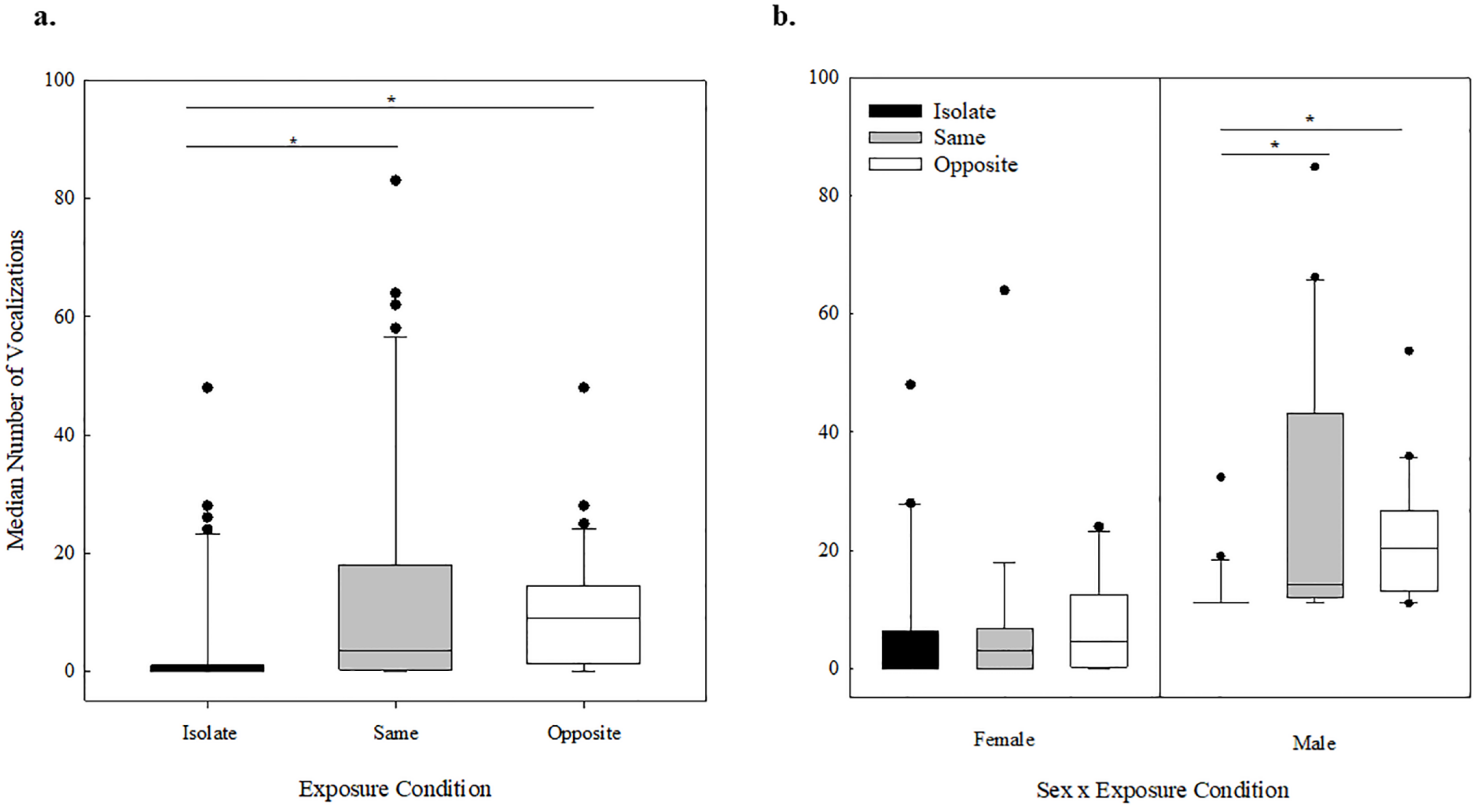
Number of vocalizations across exposure conditions. **(a)** Box plot showing the range of vocalizations produced across exposure conditions, the median (line in the box), and 95% confidence intervals. The black dots represent data points that lie outside the 10^th^ and 90^th^ percentiles. The isolate condition is shown in black, the same-sex condition is shown in gray, and the opposite sex condition is shown in white. The * represent significantly different conditions. **(b)** Box plot showing the range of vocalizations produced across exposure conditions, the median (line in the box), and 95% confidence intervals. The black dots represent data points that lie outside the 10^th^ and 90^th^ percentiles. The left half of the figure is females and the right half of the figure is males. The isolated condition is shown in black, the same-sex exposure condition is shown in gray, and the opposite-sex exposure condition is shown in white.

Male and female mice produced USVs in all three exposure conditions. There was no significant effect of sex on the median number of USVs produced (*Z* = 1.34, *p* = .176) (Fig 4), but the number of USVs did differ across exposure conditions (H = 21.385, df = 2, *p* < .001) (Fig 5a). The number of vocalizations produced in the isolated condition differed from the same-sex condition (*p* < .001) and the isolated condition differed from the opposite-sex condition (*p* < .001), but the same- and opposite-sex exposure conditions did not differ from each other (*p* > .05). Further, the effect of exposure condition was significant for males (H = 20.742, df = 2, *p* < .001), but not for females (H = 3.564, df = 2, *p* = .168). (Fig 5b). Post hoc tests revealed that the number of vocalizations produced by males in the opposite-sex and isolate conditions differed (*p* < .001), and males in the same-sex condition differed from those in the isolated condition (*p* < .001). No other comparisons differed. In sum, the mice produced significantly more USVs after being exposed to a mouse of the same- or opposite-sex prior to recording than when recorded after a period of isolation. The mice produced the same number of USVs after being exposed to a mouse of the same sex or a mouse of the opposite sex. Finally, males and females did not differ in the overall number of USVs produced.

### Proportion of vocalization categories produced

**Fig 6:**
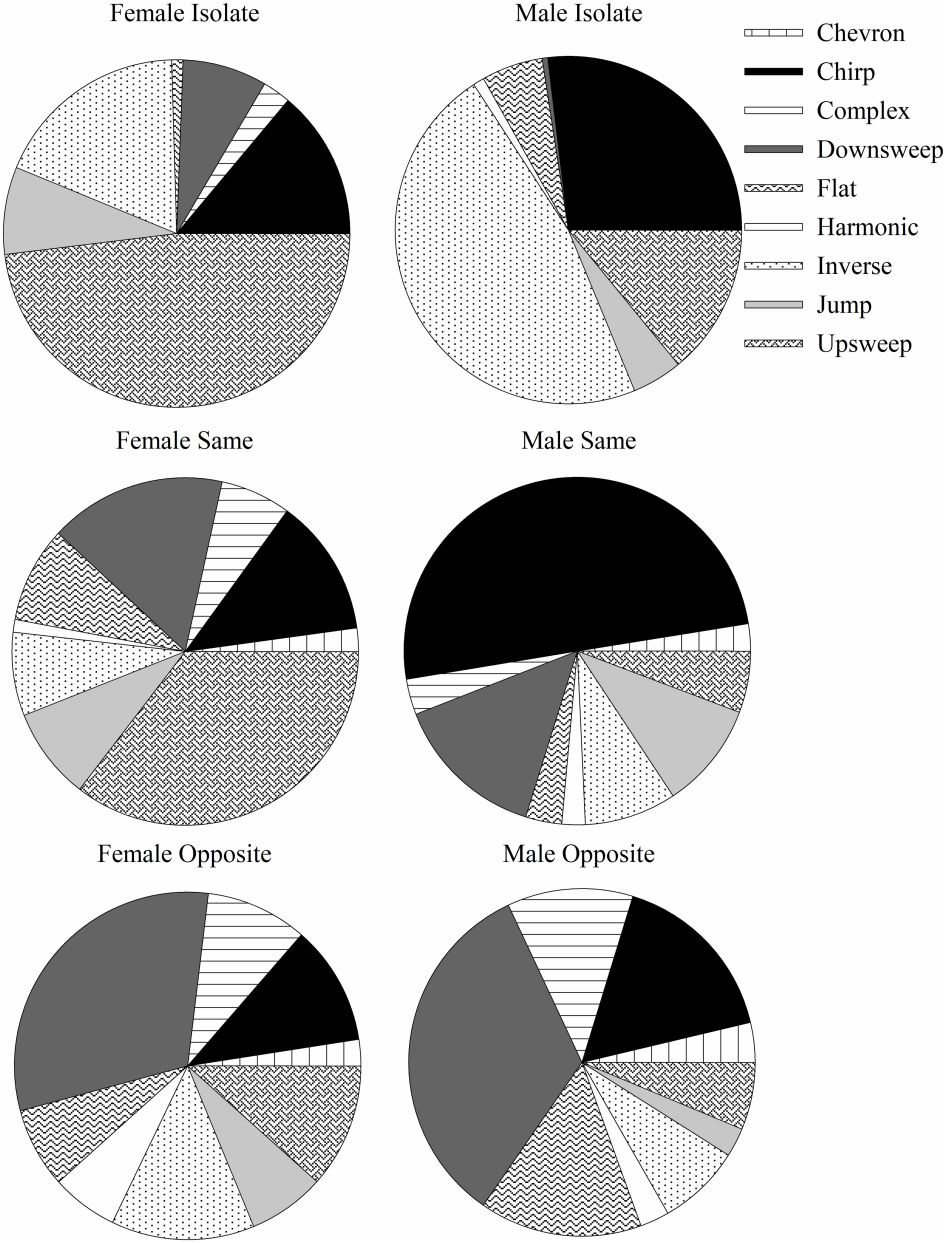
The mean proportion of USV types produced by males and females across exposure conditions. The left column is females and the right column is males. The top row is the isolated condition, the middle row is the same sex exposure condition, and the bottom row is the opposite sex exposure condition. The different colors/shadings represent the mean proportion of each of the nine different categories of ultrasonic vocalizations.

Next, the proportion of USV types produced by males and females across exposure conditions was evaluated. The means of the repertoires of vocalizations produced by animals of both sexes across the three exposure conditions are represented in Fig 6, however, because the results are not normally distributed, the statistical analysis compared median proportional probability. There were significant differences in the proportions of vocalizations produced for several USV types.

For chevron USVs, there was a statistically significant difference in the proportion of USVs produced by males across exposure conditions (H = 9.581, df = 2, *p* = .008) but not females (H = 2.976, df = 2, *p* = .226). Post hoc Mann Whitney U tests revealed that males in the opposite-sex condition differed from males in the isolated condition (*p* = .002). No other comparisons within males differed (*p* > .017). When comparing proportional production of chevrons across sexes, males and females did not differ across any exposure conditions (*p* > .017).

Like chevron vocalizations, chirps were produced in different proportions across exposure conditions by males (H = 15.622, df = 2, *p* < .001) but not females (H = .944, df = 2, *p* = .624). Post hoc Mann Whitney U tests revealed that males in the same sex condition produced significantly more chirp vocalizations than males in the isolate condition (*p* < .001). No other comparisons within males differed (*p* > .017). Comparing sex differences across exposure conditions, males and females differed in their proportion of chirps produced for same sex exposures (*p* = .003) but did not differ for other exposure types (*p* > .017).

The proportion of complex vocalizations was again different across exposure conditions for males (H = 17.949, df = 2, *p* < .001) but not females (H = 3.073, df = 2, *p* = .215). Post hoc Mann Whitney U tests revealed that the proportion of complex vocalizations produced by males in the isolate condition differed from the same-sex condition (*p* = .005) and that the opposite-sex condition differed from the isolate condition (*p* < .001). No other comparisons within males differed (*p* > .017). When looking at sex differences in proportional production, males and females did not differ (*p* > .017).

The proportion of downsweeps produced was significantly different across exposure conditions for both males (H = 16.829, df = 2, *p* < .001) and females (H = 9.437, df = 2, *p* = .009). Post hoc Mann Whitney U tests revealed that for males, the proportion of downsweeps produced in the opposite-sex and isolated exposure conditions differed (*p* < .001) and the same-sex and isolated condition differed (*p* = .007) but the same and opposite sex conditions did not differ (*p* > .017). For females, the proportion of downsweeps in the opposite-sex and isolated conditions differed (*p* = .006). No other comparisons within females differed (*p* = .017). When comparing across the sexes, none of the exposure conditions differed (*p* > .05).

For flats, the proportion of vocalizations produced across exposure conditions differed for males (H = 10.172, df = 2, *p* = .006) but not females (H = 5.446, df = 2, *p* = .066). Post hoc Mann Whitney U tests revealed that males in the opposite-sex condition produced a different proportion of flats than the isolate condition (*p* = .003). No other comparisons within males differed (*p* > .017). Additionally, no sex differences in the proportion of flats produced across conditions were present (*p* > .017).

Harmonic vocalizations differed in proportional production across exposure conditions for females (H = 12.033, df = 2, *p* = .002) but not males (H = 4.790, df = 2, *p* = .091). Post hoc Mann Whitney U tests revealed that the proportion of harmonics produced by female mice in the opposite-sex and isolate exposures differed (*p* = .005). No other comparisons within females differed (*p* > .017) and no sex differences across conditions were observed (*p* > .017).

The proportion of inverse chevrons produced across conditions did not differ for males (H = 3.659, df = 2, *p* = .161) or females (H = 3.502, df = 2, *p* = .174). As with inverse chevrons, the proportion of jump vocalizations produced across exposure conditions did not differ for males (H = 4.264, df = 2, *p* = .119) or females (H = 1.187, df = 2, *p* = .552).

Finally, the proportion of upsweeps produced across exposure conditions differed for males (H = 6.296, df = 2, *p* = .043) but not females (H = 1.122, df = 2, *p* = .571). A Mann Whitney U post hoc test showed the proportion of upsweeps produced by males in isolate and opposite-sex condition (*p* = .017) were significantly different. No other comparisons within males were significantly different (*p* > .017). No sex differences in proportional production of upsweeps were observed across exposure conditions (*p* > .017).

### Features of vocalizations

We also compared the bandwidths, durations, and peak frequencies of each USV type separately when produced by males and females following the three exposure conditions. Not all USV types were produced by the subjects in all conditions. For categories where the USV was only produced in two out of the three exposure conditions, Mann-Whitney U tests were used to compare differences in features. For categories where the USV was produced in all three exposure conditions first a Kruskal-Wallis test was used to see if the feature differed within sex and then Mann-Whitney U tests were used for post-hoc analyses to determine which conditions differed.

Chevrons were never produced by either sex following the isolated exposure condition, so comparisons were only made on the two remaining exposure conditions. There were no significant differences in the bandwidth, duration, or peak frequency of these vocalizations within the sexes (*p* > .017) or across exposure groups (*p* > .017) for chevron vocalizations. Thus, chevrons were produced similarly across the sexes and circumstances.

Like chevrons, chirps had no significant differences in the bandwidth, duration, or peak frequency of these vocalizations within the sexes (*p* > .017) or across exposure groups (*p* > .017). Chirps did not vary in features across the sexes and exposure types.

Like chevrons, complex USVs were not produced following the no exposure condition, so comparisons were made for the other two conditions only. Complex USVs differed in peak frequency across the sexes for peak frequency when males (median = 52 kHz) and females were in the same sex exposure group (p = .001). No other comparisons were significant (*p* > .017).

Downsweeps differed significantly in bandwidth for females across exposure conditions (H = 6.588, df = 2, *p* = .037). However, no comparisons were significant (*p* > .017).

Flat USVs did not differ in bandwidth, duration, or peak frequency within the sexes (*p* > .017) or across exposure groups (*p* > .017). Thus, flat vocalizations were produced similarly across sexes and circumstances.

Like the chevrons, harmonics were never produced by either sex following the isolated condition, so comparisons were only made on the two remaining exposure conditions. There were no significant differences in the bandwidth, duration, or peak frequency of harmonic vocalizations within the sexes (*p* > .017) or across exposure groups (*p* > .017). Harmonic USVs were similar across sexes and circumstances.

Inverse chevron USVs did not differ across exposure conditions for males or females (*p* > .017), however comparisons across sexes did differ for duration. Males (median = 14 ms) and females (median = 27 ms) in the same sex exposure differed (*p* = .012), as did males (median = 15 ms) and females (median = 40 ms) in the isolated exposure condition (*p* = .003). No other comparisons were significant (*p* > .017).

As with inverse chevrons, jump USVs did not differ across exposure conditions for males or females (*p* > .017), but comparisons across the sexes did differ for some conditions. For duration of jump calls, males (median = 16 ms) and females (median = 38 ms) in the same sex exposure condition differed (*p* = .004). The peak frequency of jump calls produced by males (median = 40 kHz) and females (median = 73 kHz) in the same sex exposure condition also differed (*p* = .004). No other comparisons differed (*p* > .017).

Finally, upsweep USVs produced by females differed across conditions (H = 7.006, df = 2, *p* = .03). Post hoc Mann-Whitney U tests showed that the duration of upsweeps produced by males (median = 12 ms) and females (median = 30 ms) in the isolate exposure condition also differed (*p* = .017). Additionally, the peak frequency of upsweeps produced by males (median = 59 kHz) and females (median = 78 kHz) in the same sex exposure condition differed (*p* = .001), as did males (median = 39 kHz) and females (median = 69 kHz) in the isolate exposure condition (*p* = .017). No other comparisons differed (*p* > .017).

In sum, the analyses of the USV characteristics across sexes and exposure conditions reveal that some USVs are produced similarly across the sexes and exposure conditions (chevron, chirp, flat, and harmonic), while others are produced differently across the sexes and exposure conditions (complex, downsweep, inverse chevron, jump, and upsweep). Generally speaking, most of the difference were for the durations and peak frequencies of the vocalizations produced. In calls that differed across conditions, the females always produced USVs with longer durations and higher peak frequencies than males.

## Discussion

The goal of this study was to investigate whether there are sex differences in vocalizations produced by CBA/CaJ mice following different social interactions. Results from early studies suggested female mice do not vocalize in mix-sex dyads (e.g., [40,44]). In the current study, where we recorded from mice *after* social interactions, females reliably produced vocalizations at a rate that did not differ significantly from males. Our findings are in alignment with results from Hammerschmidt and colleagues [36], although there were significant differences in the features (e.g., duration) of some, but not all, vocalizations emitted by males and females. The three different exposure types resulted in differences in the number of vocalizations produced by mice, as well as in the proportional distribution of categories of USVs produced across the sexes.

Sex and exposure condition influenced the number of USVs that mice produced. Males produced the fewest vocalizations following a one hour period of isolation compared to an hour of social experience with a same- or opposite-sex mouse. In contrast, females produced similar numbers of vocalizations following the three exposure conditions. Males did not differ from females in the number of vocalizations produced in the same or opposite sex exposure conditions. It is unclear why males and females might respond differently across the exposure conditions, but one explanation might be that isolated males are more socially motivated in these contexts than females.

Mice in this study varied their repertoire of vocal production across the different exposure conditions despite similarities in the number of vocalizations produced across most conditions. Chirp, chevron, complex, downsweep, flat, and harmonic vocalizations were produced in different proportions across exposure conditions and sexes, while inverse chevron, jumps, and upsweeps were not. Interestingly, while inverse chevrons, jumps, and upsweeps did not have statistically different proportional production across exposure conditions and sexes, these three vocalization types did have more variable feature differences than the other categories of vocalizations. That is, inverse chevrons, jumps, and upsweeps all differed in duration and peak frequency across conditions. If mice are using these vocalizations as categories for communication, it is possible that varying the number of USVs produced would not be contextually relevant, but rather, varying the acoustic properties of those signals would be more informative. For the other six categories of USVs, it might be more meaningful to increase the signals’ stereotypy.

This study is the first of its kind to show that male and female mice vary the type and features of their vocalizations in response to an immediate *previous* social exposure. Prior researchers recorded from pairs of mice where one animal was anesthetized but was still present as a control (e.g., [40]). Others recorded from mix-sex dyads with two awake mice but attributed all of the recorded USVs to the males (e.g., [2]). In contrast, mice in the present experiment were recorded individually following social interactions, enabling us to determine which mouse was vocalizing. This study illustrates the importance of considering the social or environmental context subjects experience *prior* to recording as an influential factor on vocal communication. All differences observed across males and females, across exposure conditions, and across USV features can *only* be attributed to the manipulation that took place for the one hour before the mice were recorded, as the actual recording procedures were identical across all conditions. If these vocalizations were recorded during social interactions, the effects of these conditions may have differed.

It is also important to note that there was no habituation period at the beginning of the recordings. We wanted to obtain the vocalizations produced by the mice for the five minutes immediately following the social exposure. It is likely that the effect of exposure condition on vocal production may have disappeared if there was a habituation period. Mice with different personality traits, such as boldness and shyness, may have been differentially affected by this lack of habituation period.

Generally speaking, when there were sex differences in features of vocalizations, these differences were driven by females producing USVs with durations that were 22–29 ms longer or with peak frequencies 20–40 kHz higher than USVs from males. USVs have been inferred to be simply a byproduct of breathing [47] and air moving through the larynx [48]. A potential explanation for differences in frequencies of vocal signals across the sexes might be body size differences between males and females. However, males and females produced USVs with similar peak frequencies in all categories except for upsweeps. If this difference in peak frequency was observed for all vocalization types, body size would be a plausible explanation for the differences. Instead, it is more likely that, if these vocalizations are important for mating, the frequency content of particular USV types may be essential for determining the sex of a potential mate.

This study adds to what is known about context specificity of mouse vocalizations and understanding the categories of vocalizations that mice produce. Previous research on context specificity of mouse USVs has revealed that mice vary the production of their vocalizations at different stages in the mating sequence [2], in pleasant versus aversive environments [27], and in response to awake versus anesthetized stimulus animals [34,40]. Researchers suggest that both wild and laboratory mice use vocalizations as a means of social recognition and to aid in courtship displays [6]. Based on these earlier studies, it is clear that the social or environmental context during recording influences the production of USVs by mice. This study expands on that idea and demonstrates that the conditions mice experience prior to the recordings also affect vocal production. Social exposures lasting one hour led to differences in USV production by later isolated mice. This means that the behavioral context examined in vocal production experiments has a broader timeline than was previously known, and it should be investigated in more detail in future studies.

Genetics also plays a role in the rate of production, duration, and frequency of vocalizations produced by mice [17]. Wild and laboratory strains of mice produce USVs differently, with laboratory animals emitting less diverse repertoires of USVs with less variability in acoustic features [18]. While many different mouse strains possess the ability to discriminate between vocalizations of siblings and strangers [3] and prefer vocalizations produced by mice of strains different than their own to avoid inbreeding [26], these vocalizations have not been cataloged across different social or environmental contexts in all of the commonly used laboratory strains of mice. When comparing the categories of vocalizations produced by different strains, B6 mice have been shown to produce more jumps relative to BALB, KJR, and ICR mice [26]. BALB mice produce more harmonic vocalizations, KJR mice produce more flat vocalizations, and ICR mice produce more downsweeps. CBA/CaJ mice produce similar USV types as these strains, but they change their repertoire in response to changes in the social context. For example, following isolation, male mice produce approximately 50% inverse chevron vocalizations, and females produce approximately 50% upsweep vocalizations. These proportions are directly related to the preceding social condition, however, because following opposite-sex exposures males produce only about 20% inverse chevron vocalizations, and females produce only about 20% upsweep vocalizations. Thus, researchers may attribute USV production differences across mouse strains to genetics, while they should also be considering differences in the recording procedures across experiments. Without identical recording procedures, comparisons of vocalization repertoires among mouse strains is difficult.

It has been noted that housing conditions may play a role in vocal production [36], where mice who were isolated for more than a day responded differently to intruders than mice who were only briefly moved to isolation. All experimental subjects in the current study were chronically socially, but not acoustically, isolated from all other mice. The two types of social exposures in this experiment were the only two hours during which experimental animals had social contact with another animal. This is the standard housing condition for many laboratories, but is something that may further affect the vocal production of the mice. It is possible that the response to the social exposures or isolation would differentially affect animals that are usually housed in groups.

## Conclusions

Both male and female CBA/CaJ mice produced numerous USVs after exposure to a same- or opposite-sex mouse compared to after a period of acoustic isolation. Our results suggest that the methodology for eliciting vocalizations needs to be considered when analyzing the vocal repertoire of mice. Researchers conducting future studies must also consider the contribution of male and female mice to vocalizations recorded in mixed-sex dyads when both mice are awake and behaving. Finally, the present findings clearly suggest that the housing and previous contextual situation mice encounter prior to recording sessions can differentially influence the vocal behavior of male and female mice.

## Supporting information

S1 Table All data for current study. The numbers of calls produced by each subject, separated by sex and condition are shown in the “Number” tab. The bandwidths, peak frequencies, and durations of each call produced by each subject in each condition are shown in the “Category Features” tab. The proportion of calls produced by each subject in each condition are shown in the “Category Proportion” tab.
(XLSX)
